# Exploring the Other Side of Medication: A Patient Interview Study on Anti‐PCSK9 Monoclonal Antibodies

**DOI:** 10.1155/cdr/8814045

**Published:** 2026-06-16

**Authors:** Ting Zhang, Doudou Li, Yanmei Ning, Jiana Shi, Ying Hu

**Affiliations:** ^1^ Department of Pharmacy, Clinical Pharmacy Center, Zhejiang Provincial People′s Hospital, Affiliated People′s Hospital, Hangzhou Medical College, Hangzhou, Zhejiang, China, hznu.edu.cn; ^2^ School of Pharmacy, Hangzhou Normal University, Hangzhou, Zhejiang, China, hznu.edu.cn

**Keywords:** anti-PCSK9 monoclonal antibodies, Colaizzi analysis method, qualitative study, treatment adherence

## Abstract

**Background:**

Proprotein convertase subtilisin/kexin type 9 (PCSK9) inhibitors demonstrate significant lipid‐lowering effects, but the widespread use of anti‐PCSK9 monoclonal antibodies remains limited.

**Purpose:**

This study is aimed at understanding patients′ real‐world experiences with anti‐PCSK9 monoclonal antibodies from a subjective perspective and identifying key factors influencing medication adherence.

**Methods:**

A qualitative research methodology was employed, involving face‐to‐face semistructured in‐depth interviews with 24 individuals who had previously used anti‐PCSK9 monoclonal antibodies. The interviews were guided by a thematic framework approach and recorded in their entirety. Posttranscription, Colaizzi′s phenomenological analysis method was utilized to systematically collect and interpret patient‐reported subjective experiences.

**Results:**

Among those continuing treatment, the most common motivating factors were the anti‐PCSK9 monoclonal antibodies′ favorable efficacy and fewer adverse reactions. Among those discontinuing treatment, the primary reasons for stopping were high medication costs (62.5%), insufficient recognition of the necessity for continued use (62.5%), and unwillingness to use injection administration (68.75%). The underlying determinants of patients′ behavioral patterns can be elucidated and mitigated by applying the health belief model and the theory of planned behavior.

**Conclusion:**

Research findings indicate that patients′ willingness to use medication is primarily influenced by three factors: perceived good efficacy, health awareness, and fear of needles and economic burden. We are aimed at enhancing long‐term clinical benefits for patients and improving treatment adherence, establishing education about drug efficacy and necessity, promoting innovation of nasal spray or long‐acting implant formulations, and achieving precise medical insurance coverage for high‐risk populations.

**Trial Registration:**

ChiCTR registration number: ChiCTR2500101339

## 1. Introduction

Atherosclerotic cardiovascular disease (ASCVD) represents a severe manifestation of cardiovascular system disorders, potentially leading to serious consequences such as myocardial infarction, stroke, or even death [1]. Statins serve as the cornerstone of clinical lipid‐lowering therapy, reducing ASCVD events and lowering LDL‐C [2]. However, some patients remain intolerant to statin therapy [3]. In recent years, proprotein convertase subtilisin/kexin type 9 (PCSK9) inhibitors, exemplified by evolocumab and alirocumab, have emerged as novel lipid‐lowering agents. Used alone or in combination with statins, they demonstrate significant efficacy in preventing cardiovascular events [4, 5]. Currently, five monoclonal antibodies are most commonly used in clinical practice in China, with evolocumab and alirocumab being the most widely applied. Trials have confirmed [6, 7] that anti‐PCSK9 monoclonal antibodies significantly lower LDL‐C and reduce the risk of ASCVD events.

Despite clinical guidelines recommending anti‐PCSK9 monoclonal antibodies for high‐risk patients [8], a study [9] shows that there is bias in the distribution of anti‐PCSK9 monoclonal antibodies adherence in clinical practice, with a considerable portion of patients receiving only one injection. The actual application of anti‐PCSK9 monoclonal antibodies is limited by multiple factors, leading to limited widespread use [8, 10–14]. Existing research has primarily focused on evaluating the drug′s efficacy and safety [15–17]. However, practical concerns such as financial burden can easily trigger medication‐related anxiety among patients. Strict reimbursement and prescribing limitations set by national policies also influence medication use to some degree. For instance, in certain countries, prescriptions are limited when LDL‐C levels fall below a specific threshold [18, 19]. In China, although there are no medical prescription restrictions, because these drugs are expensive, China′s medical insurance system covers multiple types of coverage, and not all insurance plans cover them. Furthermore, patients exhibit significant variations in educational attainment and operational capabilities. Some patients opt to receive drug injections at medical institutions, whereas others who meet the necessary conditions prefer self‐administration in a home setting. To date, systematic exploration of patients′ subjective experiences remains relatively scarce.

This study adopts a multidimensional physiological–psychological–social perspective and employs qualitative research methods to deeply explore patients′ authentic experiences and behavioral barriers during anti‐PCSK9 monoclonal antibodies use. Through in‐depth face‐to‐face interviews [20], patient data was collected to listen to their subjective expressions regarding treatment efficacy, observe changes in body language and facial expressions to analyze underlying emotional responses, and thoroughly investigate patients′ perceptions of drug efficacy, experiences of side effects, and psychological feelings.

The health belief model (HBM) and the theory of planned behavior (TPB) are two cognitive‐behavioral frameworks that are foundational in the field of health psychology. They systematically explain and predict individuals′ health‐related behaviors, providing a theoretical basis for developing effective behavioral intervention strategies. The HBM [21] emphasizes that patients′ perception of disease threat, judgment of the benefits of treatment, and perceived barriers together determine their health behaviors. The TPB focuses on the predictive role of behavioral attitudes, subjective norms, and perceived behavioral control on behavioral intentions [22]. These two theories are widely used in chronic disease management and clinical research, helping to deeply understand individuals′ health decision‐making processes and behavior patterns. We hope to incorporate the HBM and the TPB to explain patient behavior and provide corresponding medication guidance.

By analyzing the positive or negative impacts of anti‐PCSK9 monoclonal antibodies use on patients′ daily quality of life and disease management burden, this research provides scientific evidence for optimizing medication strategies, enhancing treatment adherence, and improving patient quality of life. It simultaneously advances the development of patient‐centered healthcare models.

## 2. Materials and Methods

### 2.1. Study Design

This study intends to analyze adult patients who have been treated with anti‐PCSK9 monoclonal antibodies through a qualitative study and conduct face‐to‐face interviews with them [23]. We are aimed at comprehensively understanding the patients′ perceived efficacy, experience of side effects, and convenience of use during the use of anti‐PCSK9 monoclonal antibodies, and at exploring the influencing factors affecting the patients′ willingness to use anti‐PCSK9 monoclonal antibodies, in order to optimize the patients′ adherence to the treatment, and thus to promote the better development and promotion of anti‐PCSK9 monoclonal antibodies.

### 2.2. Study Sites and Participants

Interviews were conducted at Zhejiang Provincial People′s Hospital from April to May, 2025, and included 24 patients in the Department of Cardiovascular Medicine, aged ≥ 18 years old with a confirmed diagnosis of ASCVD [24] defined according to clinical criteria, including documented history of coronary artery disease (myocardial infarction or revascularization), cerebrovascular disease (ischemic stroke or transient ischemic attack), or peripheral arterial disease, or familial hypercholesterolemia (FH). FH was diagnosed according to the Dutch Lipid Clinic Network (DLCN) criteria, with patients classified as having FH if the DLCN score was ≥ 6 points (definite or probable FH) [25]. Patients have received treatment with anti‐PCSK9 monoclonal antibodies (evolocumab or alirocumab) as prescribed by physicians. Patients treated with inclisiran were not included in this study. For each patient, the specific type, dosage, and dosing interval of anti‐PCSK9 monoclonal antibodies were recorded.

The study protocol was approved by the Ethics Committee of Zhejiang Provincial People′s Hospital and was conducted in accordance with good clinical practice and applicable regulatory requirements. All eligible participants provided written informed consent before being scheduled for an interview, and patients voluntarily participated in the study and signed it, being able to clearly express their feelings and opinions.

### 2.3. Definitions of Persistence and Discontinuation of Therapy

Patients with continued medication should meet the requirement that there is no medication gap of ≥ 60 days from the last supply day of one prescription to the next prescription fill [26]. Patients with discontinued medication are those with treatment intervals of ≥ 90 days and no anti‐PCSK9 monoclonal antibodies prescription or injection record [27]. This interval was verified through pharmacy records, medical record review, and patient interviews.

### 2.4. Ethical Considerations and Consent to Participate

The study was approved by the Research Ethics Committee of Zhejiang Provincial People′s Hospital (Ethics No. KY2025038), and informed consent was obtained from each patient before data collection.

### 2.5. Qualitative Research Methods

We employed qualitative descriptive analysis methods [28] and carried out semistructured, in‐depth interviews. These interviews were facilitated by healthcare professionals with training in qualitative research methods. Each interview lasted roughly 15 min, was audio‐recorded, and then transcribed word‐for‐word. Data collection and analysis proceeded iteratively. Interviews were transcribed verbatim and analyzed thematically using an inductive approach. Recruitment continued until thematic saturation was achieved, defined as the point at which no new codes or themes emerged from successive interviews. Saturation was assessed through ongoing discussion among the research team after each set of three to five interviews. After 24 interviews, we observed that subsequent interviews yielded no new insights relevant to the research questions, and saturation was therefore considered reached. The interview guide, which was based on a literature review and the researchers′ clinical insights, was designed to explore patients′ actual experiences with anti‐PCSK9 monoclonal antibodies, particularly their perspectives on the treatment′s effectiveness, side effects, and convenience. Specific interview details can be found in Table S1.

### 2.6. Data Analysis

Audio recordings were transcribed verbatim by two interviewers manually within 24 h subsequent to each interview. Subsequent to transcription, seven transcripts were randomly selected from the 24 for accuracy verification. Data analysis was conducted employing Colaizzi′s seven‐step content analysis method [29], which comprises the following sequential steps: (1) reading and re‐reading each transcribed text to obtain a comprehensive understanding of the content; (2) extracting significant statements pertinent to the research topic from the transcribed material; (3) interpreting the meanings embedded within these significant statements; (4) organizing the derived meanings into thematic clusters; (5) synthesizing the findings into a holistic description; (6) delineating the underlying structure of the phenomenon under study; and (7) subjecting the results to participant validation by feeding them back, thereby enhancing the study′s validity. This study incorporated a member‐checking strategy, involving validation of the findings′ accuracy and translation with the participants. Two authors independently reviewed the identified themes assessing internal consistency and external distinctiveness, documenting pertinent data extraction. In instances where consensus was not achieved, a third researcher was consulted to mediate. The final themes were subsequently discussed and ratified by the full research team.

## 3. Results

Twenty‐four participants completed the qualitative study. Eight participants were on anti‐PCSK9 monoclonal antibodies therapy at the time of interview, and 16 were off therapy. The continuation group had a mean age of 67.63 ± 8.88 years, whereas the discontinuation group was 66.56 ± 10.41 years, with no statistically significant difference between the groups (*p* = 0.759). Regarding age group distribution, there was also no significant difference observed between the two groups (*p* = 0.682). A total of 81.25% of patients in the discontinuation group were male. Except for education level, the two groups were balanced and comparable in baseline characteristics such as age, gender, occupation, living arrangement, and injection site. The specific demographic characteristics of the participants are shown in Table 1.

**Table 1:** Demographic characteristics of participants.

	Continuers (*n* = 8)	Discontinuers (*n* = 16)	*p*value
Age (years)	67.63 ± 8.88	66.56 ± 10.41	0.759
Age group			0.682
< 60 years	2	2	
60–70 years	3	6	
> 70 years	3	8	
Gender			0.112
Male	4 (50.00)	13 (81.25)	
Female	4 (50.00)	3 (18.75)	
Educational attainment			0.036
Elementary school	6 (75.00)	3 (18.75)	
Middle school	2 (25.00)	5 (31.25)	
High school	—	4 (25.00)	
College	—	4 (25.00)	
Occupation			0.646
Enterprise employees	5 (62.50)	6 (37.50)	
Retiree	2 (25.00)	8 (50.00)	
Farmer	1 (12.50)	2 (12.50)	
Whether living with children			0.074
Yes	3 (37.50)	12 (75.00)	
No	5 (62.50)	4 (25.00)	
Hospital injection	2 (25.00)	10 (62.50)	0.083
Injections at home	6 (75.00)	6 (37.50)	

A total of 24 patients were enrolled in this study. The two groups showed no significant differences in medication indications, coronary heart disease severity, left ventricular ejection fraction, concomitant medication approaches, or the prevalence of comorbidities like hypertension and diabetes (*p* > 0.05), indicating comparable baseline clinical characteristics. With respect to therapeutic regimens, patients received either evolocumab at a dosage of 140 mg administered biweekly or alirocumab at 75 mg administered biweekly. Specific clinical characteristics and treatment parameters are delineated in Table 2. The two groups had no significant differences in most clinical characteristics and treatment backgrounds, and discontinuation behavior may have been more influenced by nonclinical factors such as individual tolerance or socioeconomic factors.

**Table 2:** Clinical characteristics and treatment details.

	Continuers (*n* = 8)	Discontinuers (*n* = 16)	*p*value
**Type of drug, n (%)**			0.076
Evolocumab (140 mg Q2W)	8	11	
Alirocumab (75 mg Q2W)	0	5	
**Reasons for using anti-PCSK9 monoclonal antibodies,** **n** **(%)**			0.759
ASCVD	5	11	
Familial hypercholesterolemia	3	5	
**Lipid profile**			
LDL‐C before injection (mg/dL)	2.58 ± 0.98	3.42 ± 1.55	0.178
LDL‐C at last injection (mg/dL)	0.97 ± 0.72	1.62 ± 0.68	0.057
**Severity of coronary artery disease**			0.776
Mild	1	4	
Moderate	3	5	
Severe	4	7	
**Cardiac function**			
LV ejection fraction (%)	60.00 ± 4.00	63.06 ± 7.94	0.124
**Prior lipid-lowering therapy, n (%)**			0.733
None	1	1	
Statin therapy	2	4	
Ezetimibe therapy	0	2	
Statin and ezetimibe therapy	5	9	
**Comorbidities, n (%)**			
Hypertension	7	10	0.204
Diabetes mellitus	1	3	0.699
Cardiovascular disease	4	9	0.772
Cerebrovascular disease	1	2	1.000

After a thorough comparison and summary of the interview data, several major themes were identified and summarized: (1) the main facilitators of continued use of anti‐PCSK9 monoclonal antibodies, (2) the main reasons for discontinuing the use of anti‐PCSK9 monoclonal antibodies, and (3) the areas in which patients would most like to see improvements in their use of anti‐PCSK9 monoclonal antibodies.

### 3.1. Theme 1: Main Facilitators of Continued Use of Anti‐PCSK9 Monoclonal Antibodies

Among participants (*n* = 8) who continued treatment with anti‐PCSK9 monoclonal antibodies, the most common facilitator was the excellent efficacy of treatment with the drug. Some participants who had previously taken statins experienced side effects and switched to anti‐PCSK9 monoclonal antibodies with significant efficacy, or after trying other lipid‐lowering medications that did not work, found that anti‐PCSK9 monoclonal antibodies were effective in controlling lipid levels. Lipid levels vary among different participants; medication frequency can be adjusted based on each patient′s specific condition. The verbatim transcripts of the interviews with those who continued treatment with anti‐PCSK9 monoclonal antibodies are as follows:

“The use of anti‐PCSK9 monoclonal antibodies has been effective, I go to the hospital every time for injections and my lipids are reduced and under control.” *(Participant 12)*

“I have been suffering from hyperlipidemia for more than 2 years, before taking statins for lipid lowering there were side effects such as pain, swelling, etc. I started to use anti‐PCSK9 monoclonal antibodies under the doctor′s advice, and I inject myself at home every three weeks, and my blood lipids are under better control, and I am willing to use anti‐PCSK9 monoclonal antibodies for lipid lowering in the long term.” *(Participant 1)*

“After taking statins I felt very weak and then stopped taking them, now I do not feel that way with anti‐PCSK9 monoclonal antibodies.” *(Participant 14)*

“Previously taking other lipid‐lowering drugs were not very effective the use of anti‐PCSK9 monoclonal antibodies after the blood lipids are under good control, rather than taking medication every day, I prefer once every 3 weeks of injections.” *(Participant 24)*

### 3.2. Theme 2: Main Reasons for Discontinuing Anti‐PCSK9 Monoclonal Antibodies Use

#### 3.2.1. Lack of Medication Awareness and Long‐Term Use

During the course of anti‐PCSK9 monoclonal antibodies treatment, 16 participants discontinued their medication. The most common reasons for discontinuation were that most participants lacked the awareness to continue dispensing the medication after it had been used up and failed to recognize the importance of long‐term continuous use, and some participants stopped taking the medication on their own when they saw that their lipid profiles had returned to normal. Interviews with anti‐PCSK9 monoclonal antibodies discontinuers were recorded verbatim as follows:

“Due to work, twice weekly shots of alirocumab are often forgotten, leading to a gradual cessation of use of the medication, and it is only when I feel unwell and go to the hospital for a check‐up that I remember to be on the medication again.” *(Participant 4)*

“I was using it when I was hospitalized and stopped using it after I was discharged from the hospital when I ran out of the dispensed medication, I probably used Evolocumab 3‐4 times, and it was a bit of a hassle to make a trip to the hospital and I did not think about getting it dispensed and then continuing to use it.” *(Participant 5)*

“The medication was stopped when I went to the hospital to check that the lipid index was within the normal range.” *(Participant 15)*

#### 3.2.2. High Price of Drugs

Factors such as the high price of the drug and the inability of health insurance to fully cover patients′ reimbursement for the drug make patients face greater financial pressure, leading to hesitation or even cessation of dispensing the drug even if the drug is efficacious. The following is a transcript of an interview with an anti‐PCSK9 monoclonal antibodies discontinuer:

“The anti‐PCSK9 monoclonal antibodies is a bit expensive, and I did not continue using it after my lipids reached the standard.” *(Participant 23)*

“Because my liver function is not very good can not take oral medication started to use anti‐PCSK9 monoclonal antibodies for injection treatment, but the price of this drug is too expensive, my health insurance can not be reimbursed, the price of the drug will cause a burden to me, later after the recovery of liver function do not want to take injections.” *(Participant 17)*

#### 3.2.3. Injectable Medications and Side Effects

Anti‐PCSK9 monoclonal antibodies need to be administered through injections. Some patients face difficulties with the injection process, have a fear of needles, or are concerned about the impact of side effects, leading to reduced compliance with their medication. Below is a verbatim transcript of interviews with patients who have discontinued anti‐PCSK9 monoclonal antibodies:

“After I ran out of the medication I was unaware that I needed to continue to dispense the medication for long term use, there was no one in the house with me, my ears could not hear to determine if I had finished injecting the medication, and it was a hassle to go back and forth to the hospital to get the injection.” *(Participant 9)*

“I had significant pain when I used Evolocumab for injections, and my sleep quality was affected after using it, and because I am more sensitive to pain myself, I did not choose to dispense the drug again after I ran out of the medication I had been prescribed.” *(Participant 8)*

### 3.3. Theme 3: What Patients Would Most Like to Improve With Anti‐PCSK9 Monoclonal Antibodies

Patients′ willingness to take medication is diminished by high costs, whereas a lack of health awareness and fear of needles also hinder consistent drug adherence. Patients expect further optimization regarding economic factors, drug dosage forms and medication adherence—aligning with the primary barriers affecting their medication use. Detailed information is presented in Table 3.

**Table 3:** Participants′ medication feedback and willingness to use.

Theme	Representative interview feedback	Total number of interviewees (*n* = 24)
**Financial burden**	*“*I am an ordinary farmer with no stable income source. The cost of medication relies primarily on support from my children… I hope that by lowering drug prices or expanding health insurance coverage, the financial burden of long‐term treatment can be eased.*”* (Participant 24)	20
	“The cost of injectable treatments is currently quite high… If the price of injections could be brought closer to that of oral medications, I would still be willing to continue using anti‐PCSK9 monoclonal antibodies.” (Participant 23)	

**Injection inconvenient**	“Typically, people go to the hospital for medication injections, but the trip to the hospital is rather inconvenient.” (Participant 12)	14
**Lack of medication awareness**	After finishing the medication, I did not know I needed to continue taking it, so I did not go back to get a refill. (Participant 22)	10

**Optimized dosage form**	“Getting prescriptions filled at community hospitals requires injection certificates, and going back and forth to our hospital for refills is quite inconvenient… If there were longer lasting medication that could reduce the number of times I need to visit the hospital, I would be willing to use it..” (Participant 22)	15
	“I am afraid of the injection method… If more convenient dosage forms become available in the future, I would prefer that option.” (Participant 12)	

## 4. Discussion

**Figure 1:**
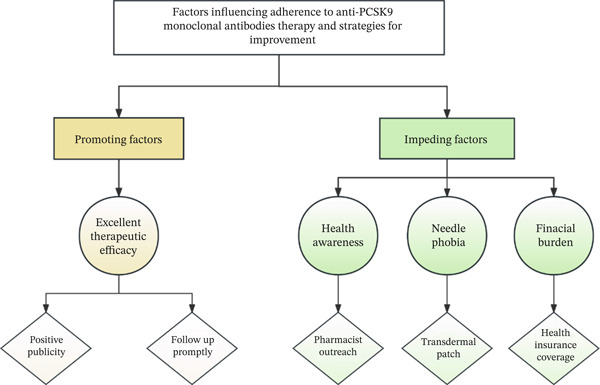
Factors affecting anti‐PCSK9 monoclonal antibodies treatment adherence and improvement strategies.

Anti‐PCSK9 monoclonal antibodies are primarily used to lower LDL‐C [30] and reduce the risk of cardiovascular events. Randomized controlled trials have confirmed that these drugs significantly reduce LDL‐C levels in patients, with their efficacy widely recognized by patients [31–33]. Our study found that favorable treatment outcomes are a key positive factor in maintaining patient adherence. However, lack of health awareness and fear of needles hinder sustained medication use, whereas most patients discontinue the drug due to financial constraints, indicating areas for improvement. Specific findings are illustrated in Figure 1.

Patients are willing to continue using anti‐PCSK9 monoclonal antibodies because they perceive the drug′s efficacy during treatment, which delivers positive and tangible health benefits. The medication is easy to administer and associated with fewer adverse reactions. This behavior aligns with the core concept of “high‐perceived benefits” in the HBM [21]. The theory is aimed at systematically constructing and reinforcing perceptions, establishing efficacy beliefs that translate the “efficacy” of the medication into significant reductions in blood lipid levels and improvements in risk assessment that patients can see and understand. The belief in the effectiveness of the medication is powerfully substantiated by data indicators, thereby enhancing the perceived benefits. Therefore, clinical settings should strengthen the promotion of the good efficacy of the medication, so that more patients persist in using it through the positive feedback of the medication. After patients use it, timely re‐examination should be conducted to allow patients to perceive the changes brought about by the efficacy, thereby increasing their willingness to continue using it.

Our study found that some patients who discontinued medication exhibited cognitive biases regarding the necessity of long‐term treatment, mistakenly believing that achieving target test values justified stopping therapy or failing to understand the need for sustained medication use. Our study population was mainly elderly, with a mean age of 63.54 ± 12.13 years. A review on medication adherence in elderly patients pointed out that cognitive impairment is a key factor for nonadherence, and older patients may have difficulty adapting to complex dosing regimens and difficulty integrating new medications into existing daily life [34, 35]. Another study highlighted the importance of health awareness in maintaining consistent behavior, noting that patients with limited knowledge about their condition and treatment rationale were significantly less likely to continue long‐term therapy [36]. This phenomenon can be explained from the perspective of the TPB [21]. Behavioral intention is determined by three factors: behavioral attitude, subjective norm, and perceived behavioral control. Patients′ cognitive biases are a comprehensive reflection of their negative behavioral attitudes, weak subjective norms, and insufficient perceived behavioral control, ultimately leading to their behavioral intention to stop medication. Based on this theoretical framework, improving patients′ medication behavioral intentions requires systematic interventions from the three core dimensions of their subjective cognition. In the future, standardized medication education programs should be established to change patients′ behavioral attitudes. Pharmacists should play a role in guiding the use of anti‐PCSK9 monoclonal antibodies to strengthen patients′ subjective norms. Additionally, a systematic follow‐up mechanism should be established to enhance perceived behavioral control. For patients with limited education who do not fully understand their prescription plan, continuous efforts should be made to reinforce their understanding of the importance of long‐term treatment.

Some participants avoided this treatment due to fear of needle injection pain, with such perceived control barriers diminishing their willingness to adhere. A systematic review and meta‐analysis found that needle phobia affects about 20%–30% of the general population and is linked to nearly 1.8 times higher risk of treatment discontinuation, independent of clinical efficacy [37]. However, some patients find the injection frequency of once or twice a month more convenient than taking medication daily. Therefore, we propose developing novel delivery methods such as nasal sprays or long‐acting implantable formulations, which could alleviate patients′ treatment fears, enhance their sense of control and confidence in execution, while retaining the advantage of a dosing frequency acceptable to suitable populations [38].

Economic studies on anti‐PCSK9 monoclonal antibodies [39] indicate that Evolocumab demonstrates cost‐effectiveness, particularly in high‐risk patient subgroups. However, most such studies focus on developed countries. In developing nations, particularly rural populations with lower incomes, economic disparities exist. Some patients exhibit low willingness to use these drugs due to financial constraints. In a PCSK9 inhibitor survey, 75% of users were over 60 years old, with out‐of‐pocket costs being the primary reason for discontinuation. Elderly patients may be particularly sensitive to high medication expenses [40]. In a study of patients receiving injectable therapies, it was found that cost‐related nonpersistence accounted for up to 40% of discontinuations in uninsured or underinsured populations [41]. Economic analyses indicate that high‐risk populations with better economic conditions could benefit from targeted health insurance coverage. Precise identification of high‐risk patients intolerant to statins or experiencing poor efficacy could enable higher reimbursement rates for these individuals. Patients who discontinue treatment due to cost face increased risks of severe complications, potentially leading to higher subsequent treatment expenditures and ultimately worsening their economic burden.

Previous research has investigated why patients stop taking statins, which mainly stem from patients′ low estimation of their own cardiovascular risk, desire to reduce medication burden, or concerns about adverse reactions [42, 43], with patients feeling confused about treatment indications due to perceived lack of professional knowledge [44]. Similarly, as a lipid‐lowering drug, this study shares similarities with statin discontinuation research; patients′ willingness to discontinue medication is influenced by cognitive biases. However, what sets our findings apart is that patients more clearly showed a lack of subjective judgment about the necessity of treatment, combined with more specific external factors such as fear of injections and economic pressure. Building on this, this study further introduces the HBM and TPB as theoretical frameworks to systematically explain patient behavior changes and propose more targeted medication guidance strategies accordingly.

Patients who stop taking PCSK9 inhibitors may not only experience poor LDL‐C control but also increased lipid variability. In China, a study showed [9] that better adherence to PCSK9 inhibitors was linked to greater LDL‐C reductions and lower LDL‐C variability, indicating that continuous treatment helps achieve both more significant lipid lowering and more stable long‐term lipid control. In a longitudinal study of 618 very high‐risk patients [45], PCSK9 inhibitor users had significantly lower mean LDL‐C standard deviation compared with statin users. Furthermore, 77.3% of patients on conventional therapy showed high variability, whereas only 17.2% of PCSK9 inhibitor patients did. These findings highlight the crucial role of continuous anti‐PCSK9 monoclonal antibodies use in optimizing both the magnitude and stability of lipid‐lowering effects. Consistent with these observations, our study demonstrated that continuous use of anti‐PCSK9 monoclonal antibodies treatment reduced the average LDL‐C level from 2.58 ± 0.98 mg/dL before injection to 0.97 ± 0.72 mg/dL at the last injection. The reduction of LDL‐C was also accompanied by reduced variability, and the standard deviations of both groups also decreased, from 0.98 to 0.72 mg/dL.

This study also has limitations. The small sample size, with only eight patients continuing treatment, restricts the robustness of statistical inferences and external validity. Additionally, the sample exhibited high homogeneity, primarily comprising individuals from Zhejiang Province. This concentration of demographic characteristics may severely limit the representativeness and generalizability of findings to broader populations. The interviews lasted approximately 15 min on average. Although this duration allowed us to capture key themes, it may be relatively short for an in‐depth qualitative exploration, potentially limiting the depth of data obtained. Furthermore, variations in age, gender, cultural background, disease severity, and economic status among individuals could influence their perceptions and acceptance of the medication. We did not collect data on patients′ perceptions of cardiovascular risk or disease severity, which may be important mediators of the relationship between economic burden and treatment continuation. Our findings on economic burden and reimbursement are grounded in the Chinese healthcare context; generalizability to other healthcare systems with different access criteria should be made with caution. Therefore, further research involving larger, more diverse populations incorporating validated risk perception instruments is necessary to validate the applicability and generalizability of the current conclusions.

## 5. Conclusion

The study, involving in‐depth interviews with 24 patients on anti‐PCSK9 monoclonal antibodies, revealed that their inclination to continue medication is significantly influenced by positive efficacy feedback, cognitive biases, fear of needles, and economic concerns. To enhance adherence to the treatment, it is crucial to amplify the positive outcomes of the drug and the need for long‐term use. Additionally, bolstering patient education and doctor–patient communication, establishing standardized educational protocols with regular follow‐ups, and emphasizing the importance of prolonged therapy are essential. Innovating in drug delivery methods, such as developing nasal sprays or long‐acting implantable formulations, will make the administration more convenient. Moreover, refining medical insurance policies to precisely target high‐risk individuals will alleviate their financial burden, improve overall outcomes, and ensure broader accessibility and long‐term benefits of anti‐PCSK9 monoclonal antibodies for a larger population.

**Nomenclature**
ASCVD: atherosclerotic cardiovascular disease
FH: familial hypercholesterolemia
LDL‐C: low‐density lipoprotein cholesterol
PCSK9: proprotein convertase subtilisin/kexin type 9

## Author Contributions

Ting Zhang and Ying Hu contributed to the study conception and design. Doudou Li and Yanmei Ning assisted in designing interview outlines. Doudou Li and Jiana Shi invited interviewed patients and assisted in collecting and organizing interview data. Ying Hu guided and supervised the research process. Ting Zhang conducted the analysis of the first transcripts and wrote the manuscript.

## Funding

This study was supported by the National Natural Science Foundation of China, Youth Science Foundation Project (No. 72204073), Zhejiang Medical and Health Science and Technology (No. 2023KY491), Zhejiang Yangtze River Delta Health Science Research Fund Project (No. 2022CSJ‐A002), and second batch of undergraduate provincial‐level teaching reform record projects in the 14th Five‐Year Plan (No. JGBA2024643).

## Disclosure

All authors have read and approved the manuscript.

## Ethics Statement

The study was approved by the Research Ethics Committee of Zhejiang Provincial People′s Hospital (Ethics No. KY2025038) and reviewed and approved in the China Clinical Trial Registry (Registration No. ChiCTR2500101339) with a registration date of April 23, 2025, and informed consent was obtained from each patient before data collection.

## Consent

The authors have nothing to report.

## Conflicts of Interest

The authors declare no conflicts of interest.

## Supporting information


**Supporting Information** Additional supporting information can be found online in the Supporting Information section. Table S1: Interview outline for participants using anti‐PCSK9 monoclonal antibodies.

## Data Availability

The dataset generated during and analyzed during the current study is available from the corresponding author on reasonable request.
