# Supplementary material for: Exploring the Other Side of Medication: A Patient Interview Study on Anti‐PCSK9 Monoclonal Antibodies
Source: Cardiovasc Ther. 2026 Jun 16;2026:8814045. doi: 10.1155/cdr/8814045 (PMC13270419; doi:10.1155/cdr/8814045)
Supplement: Supplementary file 1 — Supporting Information Additional supporting information can be found online in the Supporting Information section. Table S1: Interview outline for participants using anti‐PCSK9 monoclonal antibodies. [file CDR-2026-8814045-s001.docx]

**Supplementary Materials**

**Supplemental Table S1.** Interview Outline for Participants Using anti-PCSK9 monoclonal antibodies

| 1. How did you feel or what were your concerns when you were first introduced to anti-PCSK9 monoclonal antibodies?  2. What do you think about the overall feeling of the way the anti-PCSK9 monoclonal antibodies is used (injections) compared to the lipid-lowering medications you have used previously?  3. Did you find the way the medication was administered convenient? Did the hospital help you with the injections or did you do them yourself at home?  4. Did the use of the medication interfere with your daily schedule? |
| --- |
| **5. Are you currently still using anti-PCSK9 monoclonal antibodies? Approximately how long have you been using them? What was the main reason for stopping the drug?**   1. **Patients still using anti-PCSK9 monoclonal antibodies** 2. Would you like to continue using this medication for a longer period of time? Why? 3. What factors might motivate you to stay on it? 4. **Patients discontinuing anti-PCSK9 monoclonal antibodies** 5. What factors might make you hesitate or stop using? 6. Would you subsequently be willing to return to using the drug? Why? |
| 6. Have you experienced any side effects while using the medication? How were they dealt with? 7. What practical difficulties have you encountered while using the medication?  8. If alternative medications become available in the future, will your choices change?  9. Some patients are concerned about the cost of medications; do you have similar concerns? Do you use health insurance?  10. Does Medicare reimburse for anti-PCSK9 monoclonal antibodies? Would you be willing to use the medication for a long period of time if there were policies in place to mitigate the cost?  11. If you were to use/re-use anti-PCSK9 monoclonal antibodies in the future on a long-term basis, what would you most like to see improved in drug development?  12. What additional information about anti-PCSK9 monoclonal antibodies would you like to receive?  13. Are there any other experiences or suggestions you would like to share? |
